# The essential genomic landscape of the commensal *Bifidobacterium breve* UCC2003

**DOI:** 10.1038/s41598-017-05795-y

**Published:** 2017-07-17

**Authors:** Lorena Ruiz, Francesca Bottacini, Christine J. Boinett, Amy K. Cain, Mary O’Connell-Motherway, Trevor D. Lawley, Douwe van Sinderen

**Affiliations:** 1 School of Microbiology and APC Microbiome Institute, National University of Ireland, Cork, Western Road, Ireland; 2 Wellcome Trust Sanger Institute, Hinxton Cambridge, UK; 3 Department of Nutrition, Bromatology and Food Technology, Complutense University, Avda Puerta de Hierro s/n, 28040 Madrid, Spain

## Abstract

Bifidobacteria are common gut commensals with purported health-promoting effects. This has encouraged scientific research into bifidobacteria, though recalcitrance to genetic manipulation and scarcity of molecular tools has hampered our knowledge on the precise molecular determinants of their health-promoting attributes and gut adaptation. To overcome this problem and facilitate functional genomic analyses in bifidobacteria, we created a large Tn*5* transposon mutant library of the commensal *Bifidobacterium breve* UCC2003 that was further characterized by means of a Transposon Directed Insertion Sequencing (TraDIS) approach. Statistical analysis of transposon insertion distribution revealed a set of 453 genes that are essential for or markedly contribute to growth of this strain under laboratory conditions. These essential genes encode functions involved in the so-called bifid-shunt, most enzymes related to nucleotide biosynthesis and a range of housekeeping functions. Comparison to the *Bifidobacterium* and *B. breve* core genomes highlights a high degree of conservation of essential genes at the species and genus level, while comparison to essential gene datasets from other gut bacteria identified essential genes that appear specific to bifidobacteria. This work establishes a useful molecular tool for scientific discovery of bifidobacteria and identifies targets for further studies aimed at characterizing essential functions not previously examined in bifidobacteria.

## Introduction

Bifidobacteria are Gram-positive commensal microorganisms, widely encountered within the gut ecosystem of mammals and (certain social) insects. Extensive research has attributed health-promoting traits to some bifidobacterial strains, such as vitamin production, amelioration of certain gastrointestinal disorders and inhibition of enteropathogens through production of a range of antimicrobial compounds^1–3^. The scarcity of molecular tools for bifidobacteria, however, has hampered progress in our understanding of the specific molecular mechanisms behind their adaptation to the intestinal ecosystem, interaction with their host and possible health-promoting activities.

In recent years, various comparative genomic analyses have shed light on the evolutionary adaptation of bifidobacteria to the intestinal environment^4–7^. Furthermore, during the past decade, a number of molecular tools have been developed or adapted to certain bifidobacterial strains, generating a basic molecular tool-box including cloning and expression vectors, reporter genes and a number of inducible promoters for the control of heterologous gene expression systems^8, 9^. Successful procedures for targeted mutagenesis have also been reported, providing essential tools to perform functional genomics, which is aimed at expanding our knowledge on host-colonization factors and metabolic capabilities of this group of gut commensals^10–15^. Mutagenesis represents the most accurate route to experimentally determine if a gene is essential for a specific attribute under particular environmental conditions. However, mutagenesis procedures in bifidobacteria are still tedious, their efficiencies are highly strain dependent and frequently focus on selected target genes. In a recent report by our research group, a random mutagenesis approach based on a customized Tn*5* transposon was successfully applied to two different *Bifidobacterium breve* strains^16^, opening avenues to interrogate the functionality of bifidobacterial genes at genome level.

The genome-wide identification of essential genes, defined as those that cannot be mutated *in vitro*, or those in which a mutation reduces growth or viability under particular conditions, has been performed in other microorganisms, where screenings of mutant collections following growth under specific conditions are coupled to *en mass* genome-wide location of genomic disruptions. Such an approach allows the identification of the complete set of genes essential for a certain phenotype or bacterial attribute in a single experiment. Microarray-based approaches are used for mutant identification in TRAnsposon Site Hybridization (TraSH); while massive parallel sequencing is utilized in High-throughput Insertion Tracking Sequencing (HITS), Insertion Sequencing (In-Seq), Transposon Sequencing (Tn-seq) and Transposon Directed Insertion-Site Sequencing (TraDIS) technologies^17–23^.

Massive parallel sequencing has substantially increased the resolution power of genome-wide screening of high-density mutant collections^21–23^, thereby allowing the identification of essential genes, protein domains and intergenic regions with presumptive regulatory roles^17, 23^. Genome-wide random mutagenesis approaches are also very useful in assisting functional genome annotation and in complementing comparative genomic studies^24–27^. Such mutant library screening approaches have been used to characterize a variety of clinically relevant pathogenic microorganisms in search of novel antibacterial targets. However, so far only a small number of non-pathogenic gut commensals or potential probiotic bacteria, belonging to *Bacteroides thetaiotaomicron* and *Lactobacillus casei*, have been analyzed in this manner^18, 28^.

The aim of the current study was to further improve the applicability of the transposon mutagenesis method previously developed by our research group for *B. breve* UCC2003, by applying a genome-wide random mutagenesis approach coupled to transposon mapping by means of next generation sequencing. Analysis of transposon insertion distribution across the genome facilitated the identification of genes (designated here as essential genes) required for strain survival and normal growth on rich media under laboratory conditions. Comparative genomic analysis between the predicted essential genes and the *Bifidobacterium* core genome revealed a high level of conservation of essential genes across the genus *Bifidobacterium* and *B. breve* species, highlighting a set of highly conserved core metabolic functions, as well as a set of still uncharacterized essential functions in bifidobacteria.

## Results and Discussion

### Library preparation and TraDIS validation

In a previous publication by our research group Tn*5* transposon-mediated mutagenesis was accomplished in *B. breve* UCC2003, enabling relatively high insertion frequencies and generating single and stable transposon insertions per cell^16^. In the current work, a tetracycline resistance-conferring, Tn*5*-based transposon was employed to create a large collection of insertion mutants comprised of approximately 58,000 tetracycline-resistant colonies which were pooled from 120 independent transformation experiments (See Materials and Methods). The size of this newly constructed mutant library outnumbers the predicted number of genes of *B. breve* UCC2003 genome (1,985 genes)^29^ by about 30-fold. Assuming an even distribution of transposon insertions in the *B. breve* UCC2003 genome (2.42 Mbp), the library would be expected to contain on average one insertion every 40 base pairs. Since the smallest identified Open Reading Frames (ORFs) in this microorganism are the (predicted) tRNA genes with a size of ~77 bp^29^, this library is expected to contain mutants in most non-essential genes. In order to confirm this expectation, a transposon directed insertion sequencing (TraDIS) approach was designed and implemented in this *B. breve* UCC2003-derived mutant collection in order to identify all (or at least the vast majority) transposon insertion points. This allowed us to experimentally confirm the transposition coverage of the library and to identify essential genes based on insertion distribution across the genome. An advantage of the TraDIS approach in comparison to other massive parallel sequencing-based tools, like Tn-seq, is that TraDIS can be applied to virtually any transposon library, without the requirement of any specific sequences within the transposon^22^.

A total of 2 million reads were obtained on a HiSeq2500 platform (Illumina) and deposited in the European Nucleotide Archive (ENA) under sample accession numbers ERR877647 and ERR877656. Analysis of the generated sequences allowed us to map a high percentage of the high quality reads (98%) to the *B. breve* UCC2003 reference genome (Genbank accesion number CP000303.1). High percentage of mapped reads (usually > 90%) has previously been described as a characteristic of the mapping approach used^30^, and allowed the identification of ~46,000 unique insertions with 1 estimated insertion in every 52 bp. Of these unique insertion points, approximately 36,000 were mapped within the deduced coding regions of predicted ORFs.

**Figure 1:**
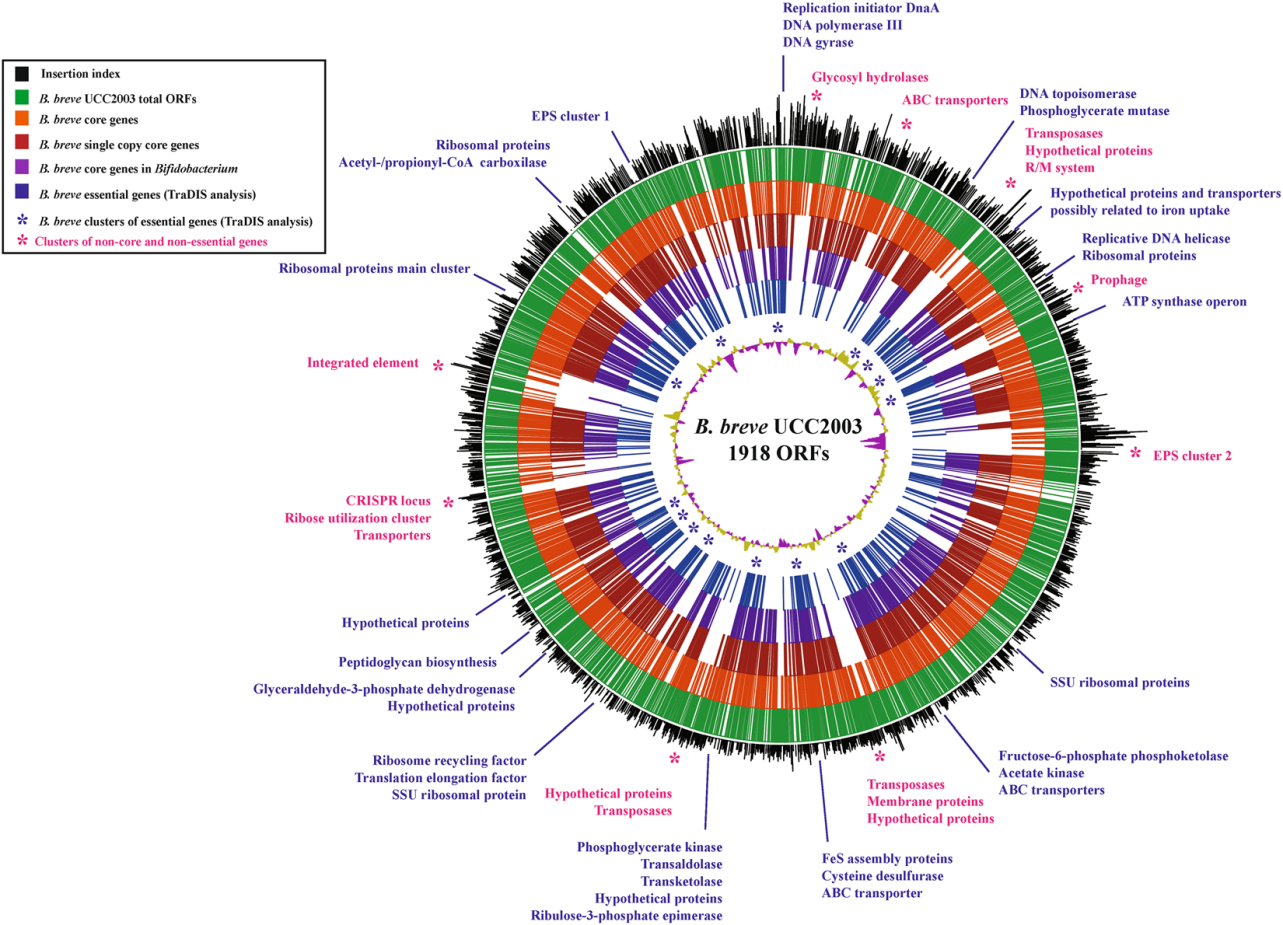
Genome-wide distribution of transposon insertions in *B. breve* UCC2003. Circular plot showing gene content, insertion distribution and gene comparison to *Bifidobacterium* and *B. breve* core genomes. Circular tracks are numbered from 1 (outer track) to 7 (inner track). Track 1 represents transposon insertion densities; track 2 indicates forward and reverse genes predicted in *B. breve* UCC2003 genome; track 3 indicates core *B. breve* genes; track 4 indicates *B. breve* core genes present in single copy; track 5 represents *B. breve* single copy core genes present in *Bifidobacterium* genus core genes; track 6 represents TraDIS predicted essential genes and track 7 represents GC bias, khaki indicates values > 1; purple < 1.

**Figure 2:**
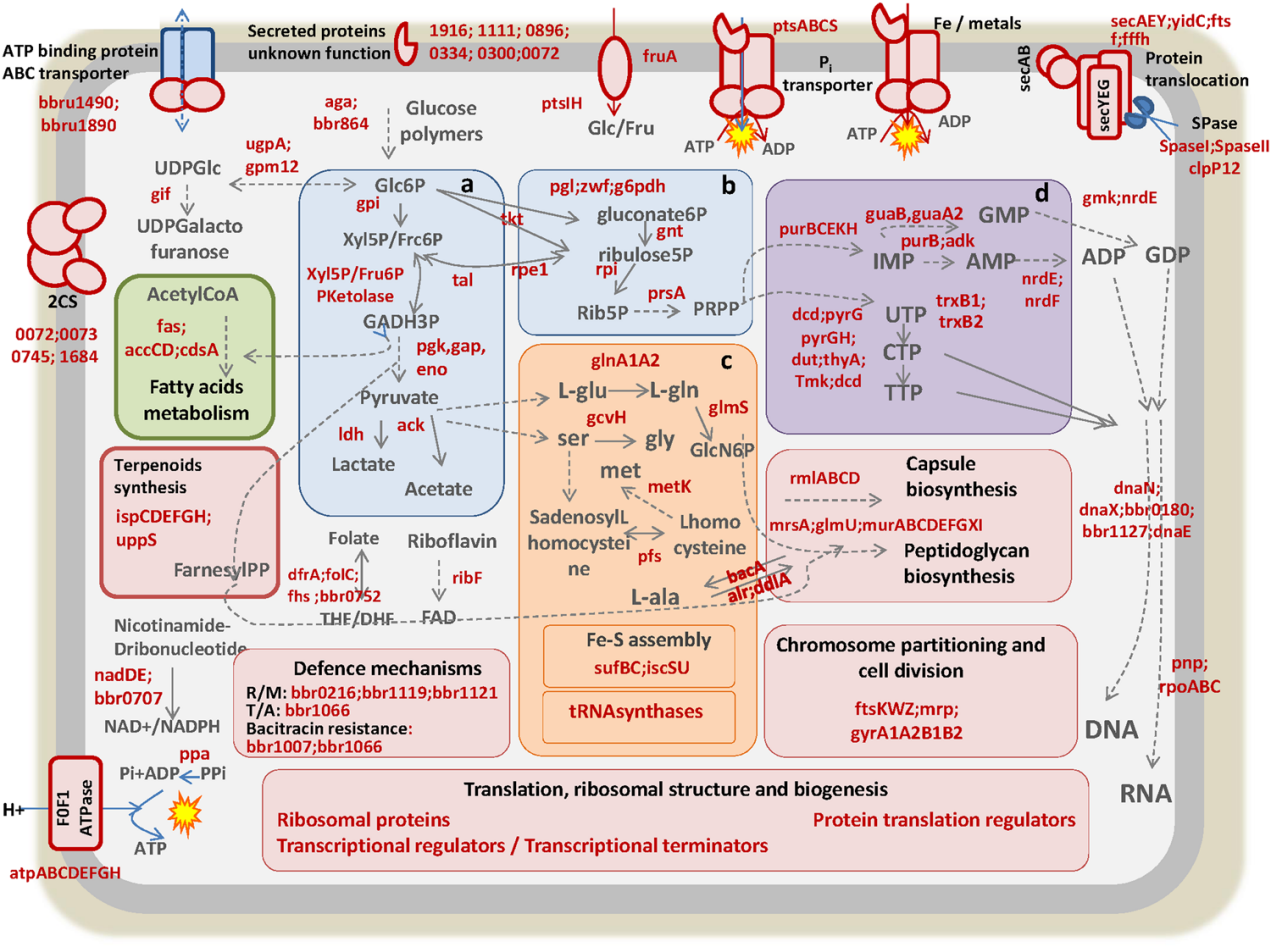
Reconstruction of essential metabolic pathways and functions, based on TraDIS-predicted essential genes in *B. breve* UCC2003. Gene names or locus tags are represented in red and metabolic intermediates in grey. Dashed arrows summarize multiple reactions. Panels A and B represent the essential central carbohydrate utilization pathways, bifid shunt and pentose phosphate pathway, respectively. Panel C represents essential steps within amino acid metabolism and biosynthesis pathways. Panel D highlights essential steps mapped onto nucleotide metabolic pathways.

High-density insertion libraries, displaying insertions at every 10 bp or even less, provide the advantage of increasing the discriminatory resolution between essential and non-essential elements^19, 31, 32^. Based on the *B. breve* UCC2003 average gene length of 1099 bp^29^ and considering the estimated frequency of one insertion every 52 bp, every non-essential gene -including small genes (<200 bp)- is expected to have been targeted by the transposon in our mutant library. Furthermore, in the case of (apparently) non-essential genes, such as Bbr_0014 or Bbr_0055, the insertion frequency was even higher (Supplementary Table S1). Indeed, a rarefaction analysis showed that our library reached saturation level (Supplementary Figure S1). In total, 1,679 genes (84.6% of all ORFs identified on the UCC2003 genome) were found to harbor at least one transposon insertion, including representatives of all functional categories (Figs 1 and 2; see ins_count column in Supplementary Table S1).

One has to keep in mind that certain gene disruptions can only be retrieved under specific environmental conditions. For this reason, the transposon mutant collection was recovered in a rich laboratory medium containing multiple carbon sources (i.e. 0.5% ribose, 0.5% glucose and 0.1% starch). These sugars are known to be metabolized through independent metabolic pathways in *B. breve* UCC2003 and, accordingly, previous works from the research group have demonstrated that mutants unable to metabolize one of those carbohydrates are still able to survive and grow using one of the other two carbon sources^10, 16, 33^. Thus, by combining multiple carbohydrates in the recovery media, the number of genes able to tolerate transposon insertions in this library and thus classified as non-essential, was maximized. Accordingly, mutants in the amylopullulanase-encoding gene *apuB* and the neighbouring maltodextrin transport cluster, known not to grow on starch as the sole carbon source, are represented in our collection. Similarly, mutants in the ribose utilization cluster are also represented in the bank (Supplementary Table S1).

In agreement with other transposon library studies, the mapped insertions were well distributed across the whole genome, with transposon densities approximately 40% higher at regions close to the chromosomal origin of replication (Fig. 1). In fact, the division of *B. breve* UCC2003 chromosome in 8 sections showed how genes located around the *ori* region possess an average of 30 insertions per gene, which decreases to 12 insertions per gene in those genes located around the *ter* region (Supplementary Figure S2a). This has previously been reported as a common phenomenon in insertion mutant libraries and has been related to the presence of a high number of replication forks in the exponential phase of growth, which is when the transposon is introduced into the cell by electroporation^34–36^. Therefore, genes close to the origin of replication are encountered in multiple copies and are thus more likely to be targeted by transposon mutagenesis. Another possible explanation for this phenomenon is related to the configuration of the genomic DNA, which would be more relaxed and accessible for transposon insertion to occur in regions close to the replication fork^17, 19^. Consistent with the latter notion, we also observed that our library shows a bias towards increased transposon frequencies in A + T rich regions, where genes display transposon densities up to 46% higher as compared to the rest of the chromosome (Supplementary Table S1, Supplementary Figs S3a and S3b), an observation in agreement with previous reports^17, 19^.

### Prediction of essential genes from TraDIS analysis

Analysis of mapped reads revealed that 1,670 protein-encoding genes (87%), which includes 7 out of the 50 identified ribosomal protein-specifying genes, 2 out of 6 rRNA-encoding genes and 7 out of the 54 identified tRNA-encoding genes harbored at least one transposon insertion. Furthermore, 255 protein-specifying genes, 4 rRNA-encoding genes and 47 tRNA-encoding genes were found not to harbour any insertion whatsoever. Several bioinformatic approaches and statistical models have been described that predict essential genes in genome wide transposon mapping experiments^23, 30, 35, 37–39^. In the current work, candidate essential genes – i.e. those that are shown not to harbour transposon insertions - in the *B. breve* UCC2003 mutant library, were predicted from the transposon insertion distribution through calculation of an insertion index per gene as previously described^30^ (Supplementary Fig. S4). Cut-off criteria to classify genes as essential were established based on the bimodal distribution of insertion indexes as previously described^30^ and indicated in the Materials and Methods section (Supplementary Fig. S4). Additional manual curation and inspection of each gene was performed in order to exclude situations of insertions not evenly distributed across the whole gene length, and thus not affecting gene function. In addition, our mapping parameters do not allow for repeat mapping and therefore, no insertions were mapped in transposons as these are expected to represent false positives, and are for this reason excluded from the essential gene list. Overall, our analysis resulted in a compiled list of (what we designate here as) essential genes, which includes 453 genes (22.8% of the total number of identified genes on the genome), including 50 that encode ribosomal proteins, 49 that represent tRNA-encoding genes and 4 being rRNA-encoding genes. The predicted percentage of essential genes is within the range reported from genome-wide surveys of essential genes in other microorganisms, which has been shown to range from 5 to 35% of the overall gene content^30–32, 40–42^.

## Functional annotation of essential genes in ***B. breve*** UCC2003

Functional analysis of predicted essential protein-encoding genes in *B. breve* UCC2003 was performed. The full list of essential genes predicted in our *B. breve* UCC2003 library, along with corresponding COG families, KEGG pathways and functional classification, are presented in Supplementary Table S2. This analysis revealed entire metabolic pathways or specific steps in particular pathways that are critical to support *B. breve* UCC2003 growth under the applied conditions. It is worth noting that our prediction of essential genes was performed straight away following the mutagenesis procedure, without any prior enrichment step recovering the mutants in liquid media. Therefore, essential genes are estimated based exclusively on the inability to recover viable mutants. Accordingly, a substantial proportion of house-keeping functions, including proteins responsible for DNA replication and transcription, manufacture of cell envelope components, such as cell wall and membrane, the Sec-dependent protein translocation pathway, and central routes for energy acquisition and conversion are abundant among TraDIS-predicted essential functions. Among the cell envelope components, it is worth highlighting that, from the two exopolysaccharide clusters encoded by *B. breve* UCC2003, only the genes encoding for the EPS cluster 1 (Bbr_1798 to Bbr_1793) were deemed essential (Fig. 1). This represents a novel finding since previous work had only inactivated and proved functionality of EPS cluster 2 (Bbr_0434 to Bbr_0451) which was not essential for *in vitro* growth, yet is required for *in vivo* colonization^43^. In addition, most genes involved in central glycolytic routes, the specific bifid-shunt^44^ and the pentose phosphate pathway, crucial for production of energy and precursors for other metabolic routes, were found to be essential (Fig. 2). Furthermore, all elements involved in ATP synthase assembly (*atpA, atpB, atpC, atpD, atpE, atpF, atpG* and *atpH*) appeared essential. In fact, ATP synthase which couples H^+^ extrusion to ATP synthesis is necessary to maintain cellular pH homeostasis, required as bifidobacteria growth leads to production of organic acids^45^ (Fig. 2, Table 1, Supplementary Table S2).

**Table 1:** COG and KEGG classification of *B. breve* essential genes.

COG Family	Pathway (KEGG)	Genes	%*
Amino acid transport and metabolism	Alanine aspartate glutamate	2	0.5%
	Amino acid related functions	3	0.8%
	Glycine serine threonine	1	0.3%
	Selenocompounds	1	0.3%
Carbohydrate transport and metabolism	Transporters	4	1.0%
	Amino sugar metabolism	1	0.3%
	Bifid shunt	3	0.8%
	Carbohydrate related functions	5	1.3%
	Carbohydrate transport and metabolism	2	0.5%
	Glycolysis/gluconeogenesis	2	0.5%
	Glycosyl hydrolases	2	0.5%
	Pentose phosphate	7	1.7%
Cell cycle control, cell division, chromosome partitioning	Cell cycle control, cell division, chromosome partitioning	6	1.5%
Cell wall/membrane/envelope biogenesis	Alanine aspartate glutamate	1	0.3%
	Amino sugar metabolism	1	0.3%
	Capsule biosynthesis	6	1.5%
	Cell wall associated functions	8	2.0%
	D-alanine	2	0.5%
	Peptidoglycan biosynthesis	9	2.3%
Coenzyme transport and metabolism	Coenzyme related functions	4	1.0%
	Cysteine methionine	1	0.3%
	Folate	4	1.0%
	Nicotinate nicotinamide metabolism	3	0.8%
	Panthotenate coA	1	0.3%
	Riboflavin utilization (energy metabolism)	1	0.3%
	Thiamine	1	0.3%
Defence mechanism	Transporters	3	0.8%
	Defence mechanism	4	1.0%
DNA, replication, recombination and repair	DNA related functions	3	0.8%
	Recombination	4	1.0%
	Repair	5	1.3%
	Replication	15	3.8%
Energy production and conversion	Citrate cycle	2	0.5%
	Energy production conversion related functions	13	3.3%
	Pyruvate metabolism	1	0.3%
Inorganic ion transport and metabolism	Transporters	12	3.0%
	Inositol phosphate	1	0.3%
Intracellular trafficking, secretion, and vesicular transport	Secretion	1	0.3%
	Secretion (sec pathway)	7	1.8%
Lipid transport and metabolism	Fatty acids biosynthesis	3	0.8%
	Lipids metabolism	5	1.3%
	Panthotenate coA	1	0.3%
	Terpenoid	7	1.8%
Nucleotide transport and metabolism	Cysteine methionine	1	0.3%
	Nucleotide related functions	1	0.3%
	Purine metabolism	9	2.3%
	Pyrimidine metabolism	11	2.8%
	Nucleotide transport and metabolism	2	0.5%
Posttranslational modification, protein turnover, chaperones	Posttranslational modification, protein turnover, chaperones	12	3.0%
RNA processing and modification	RNA processing and modification	3	0.8%
Signal transduction mechanisms	Signal transduction mechanisms	3	0.8%
	Signal transduction related functions	6	1.5%
Transcription	RNA polymerase	4	1.0%
	Transcription	1	0.3%
	Transcriptional regulator	10	2.5%
Translation, ribosomal structure and biogenesis	Ribosome	52	13.0%
	Translation	18	4.5%
	tRNA processing and modification	2	0.5%
	tRNA synthetase	22	5.5%
	tRNA-dihydrouridine synthase	4	1.0%
General function prediction only	General function prediction only	15	3.8%
Unassigned function	Unassigned function	56	14.0%
	TOTAL	400	100.0%

*Percentage of the protein encoding predicted essential genes from *B. breve* UCC2003, mapped into each COG and KEGG functional category.

With regards to specific anabolic routes, the majority of the genes involved in purine and pyrimidine synthesis were found to be essential, suggesting that *B. breve* UCC2003 relies primarily on *de novo* nucleotide synthesis. Furthermore, essential genes related to amino acid metabolism map into the biosynthesis of L-glutamate and L-glutamine which are precursors for the aminosugar metabolic pathway. Among them, the genes *glmS* (locus tag Bbr_0524) and *glnA* (locus tags Bbr_1278 and Bbr_0670) were retrieved as essential in our analysis. The product encoded by *glmS* is known to play a central regulatory role in nitrogen metabolism, catalyzes key steps for the synthesis of cell wall precursors, and has also been found essential in *Bacteroides* ^18, 32, 46^. Interestingly, our data suggests that also the presumptive transcriptional regulator of *glmS*, encoded by *glnR* (locus tag Bbr_0292), is essential. Another essential gene related to amino acid metabolism is *gcvH* (locus tag Bbr_0481) whose encoded protein participates in glycine detoxification^47^. The limited presence of essential genes in other amino acid biosynthetic routes suggests that *B. breve* UCC2003 possesses the capacity to import most required amino acids from the environment, although specific amino acid transporters have not yet been functionally characterized in this strain.

Putative inorganic ion transporters were also retrieved as essential functions, including predicted import systems for manganese, phosphate, potassium and iron. These ions are essential cofactors for many cellular enzymes and some import systems have previously been found to be essential for intestinal colonization of enteropathogens^48, 49^. Furthermore, Mn^2+^ uptake systems have been associated with oxidative stress protection in oral bacteria^50^. In fact, functions involved in the maintenance of redox homeostasis, such as glutaredoxin production and thioredoxin reductase (predicted to be encoded by Bbr_0039, Bbr_1901 and Bbr_1919 locus tags), were also found essential in our analysis. Bifidobacteria are anaerobic bacteria and are known to be exquisitely sensitive to oxygen, therefore defence mechanisms against oxidative damage are crucial. Besides, redox homeostasis regulates key metabolic functions including signal transduction mechanisms and central carbon metabolism^51^. Elements predicted to be involved in assembly of iron-sulfur clusters (*IscS, IscU, sufB, sufC, fdx*, represented by locus tags Bbr_0910, Bbr_0911, Bbr_0907, Bbr_0909, Bbr_1664), which have not been previously characterized in bifidobacteria, were also found essential in our analysis. The corresponding protein complexes are known to sense oxygen and metal availability and regulate gene expression profiles allowing rapid adaptation to environmental changes^52^, although they have mainly been studied in pathogenic bacteria. Also two genes encoding predicted orphan ATP binding proteins (Bbr_1490 and Bbr_1890), not located in proximity to any ABC transporter-encoding genes, were found to be essential in our analysis therefore suggesting they might energize crucial transport systems in the cell.

Remarkably, 53 essential genes found in this work encode hypothetical proteins with unknown functions, highlighting the limited knowledge we have on associated fundamental aspects of bifidobacterial metabolism and physiology. These findings also highlight the need for research on essential hypothetical proteins in order to define their biological function. Moreover, 11 out of the identified 453 essential genes in *B. breve* UCC2003 correspond to transcriptional regulators that have not been functionally characterized in bifidobacteria, of which nine are predicted to be involved in signal sensing and transduction, therefore representing interesting targets for future studies.

## Conserved essential genes in the ***Bifidobacterium*** and ***B. breve*** core genomes

Comparative genomics has led to important insights with regards to evolutionary drivers and pathways of bacterial taxa, allowing the identification of genes encoding functions that can be lost or acquired through horizontal gene transfer, as well as those that are seemingly very important as they are maintained across a particular taxonomic group. In this context, the concept of a core genome, consisting of the complete set of orthologous genes present in single or multiple copy shared among a group of bacteria, provides a framework to discern and understand the conserved basic biology of bifidobacteria^4–7^. Core genomes are the result of long-term natural selection, as determined by environmental conditions that facilitate the loss of non-essential genes, while at the same time preserve those functions that are critical for bacterial survival, competitiveness and persistence in their natural niche^6, 53^.

**Figure 3:**
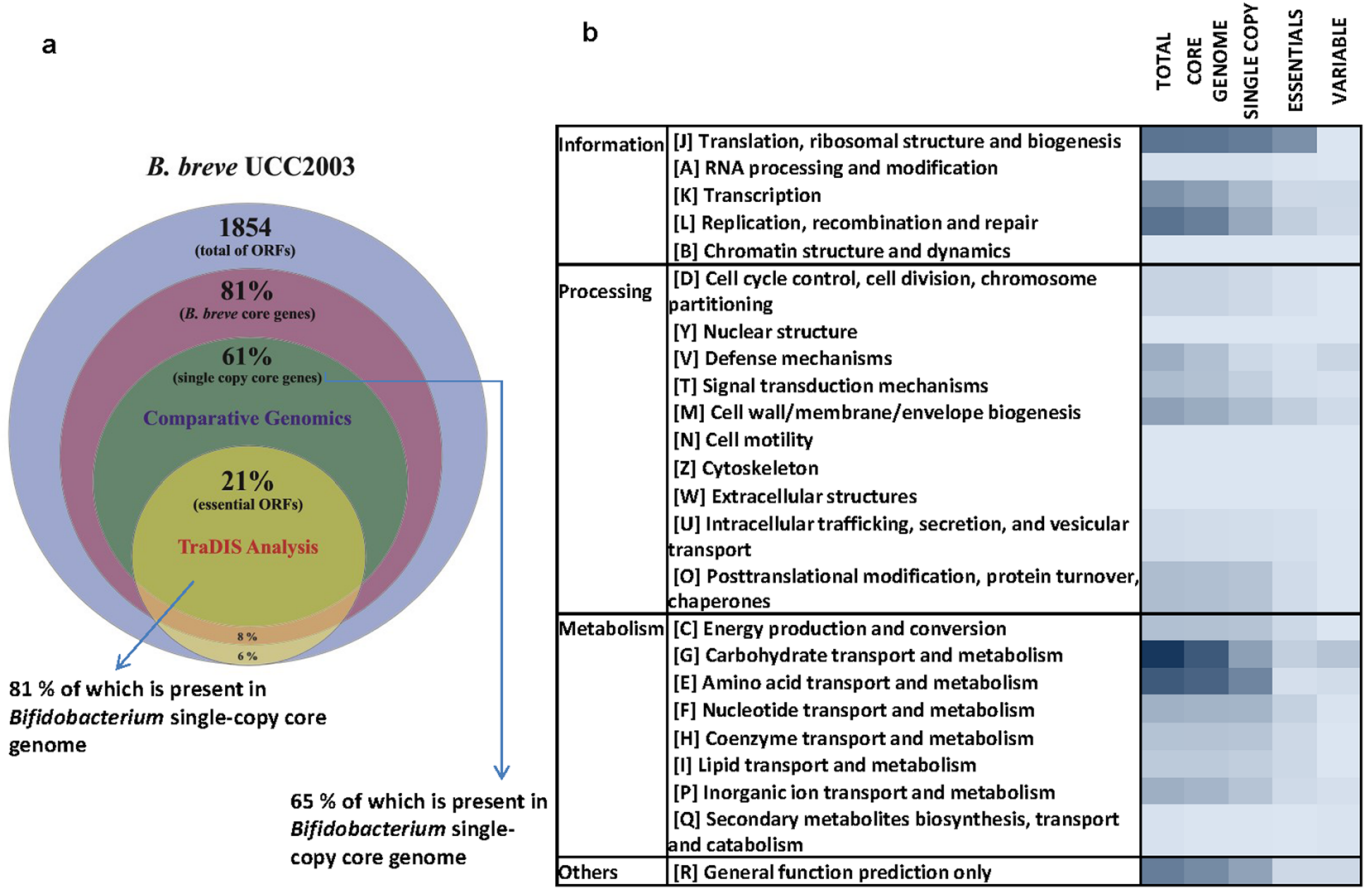
(**a**) Venn Diagram representing the overlap between the essential genes determined by TraDIS analysis for *B. breve* UCC2003, the *Bifidobacterium* and *B. breve* core genomes, as well as *B. breve* core genes present in single copy. (**b**) Heatmap representing in colour gradient the frequency of COG categories across all the different type of gene families resulting from comparative analysis of *B. breve* UCC2003 total gene content and TraDIS predicted essential genes, against the available *Bifidobacterium* genomes.

Comparative genomics coupled to transposon mutagenesis mapping has indeed been used to better define the minimal essential core genome and to reconstruct bacterial evolution, selective adaptive trait acquisition and evolutionary conservation in a number of microorganisms^25, 42, 54, 55^. For these reasons, substantial overlap between the *in silico* computed *Bifidobacterium* and *B. breve* core-genomes on one hand, and the identified essential genes from our TraDIS approach on the other, was expected. In order to test this expectation, the TraDIS-deduced essential genes of *B. breve* UCC2003 were compared to the core genomes of *B. breve* (1514 genes) and *Bifidobacterium* (929 genes), which were predicted based on 8 complete *B. breve* genomes^5^ and the genomes of 47 bifidobacterial type strains^6^, respectively. This analysis revealed that 377 out of 400 TraDIS-predicted essential genes in *B. breve* UCC2003 (94%) were also part of the *B. breve* core genome (Fig. 3a; Supplementary Table S4). This overlap is expected to occur within single-copy core genes, as they are most likely to include essential core functions evolved from a common ancestor and stably retained by selective pressure^56^. In concordance with this expectation, 75% of the *B. breve* core genome (1141 out of 1514 genes) is represented by genes present in single copy (orthologues). Remarkably, 85% of the TraDIS-determined essential genes (343 out of 400 genes) indeed belong to this *B. breve* single-copy core-genome (Supplementary Table S4). Since essential functions should not only be conserved at species level, but may also be retained at genus level, the candidate essential genes were also compared with single-copy core genes of the genus *Bifidobacterium*. This analysis revealed that indeed a large proportion of *B. breve* single-copy core genes (65%) are present in the *Bifidobacterium* core-genome, including the majority of essential genes identified by transposon insertion mapping in *B. breve* UCC2003 (81%) and representatives of all functional categories (Fig. 3a and b; and Supplementary Table 4). As the mutant library was tested under *in vitro* conditions we estimate that 43% of core genome functions in the *Bifidobacterium* genus (326 out of 748), as predicted in this work, are essential for cell viability under laboratory conditions (Supplementary Table S4). This set of genes comprises functions belonging to most COG functional categories, though in different proportions, yet is particularly enriched in house-keeping functions, such as those involved in DNA maintenance, replication, transcription and translation, in the biosynthesis of cell envelope components, and in central metabolic pathways for energy generation and metabolite conversion functions (Table 1, Fig. 3b; and Supplementary Table S4). This is in agreement with data from other microorganisms and supports the high level of conservation of a range of house-keeping functions^41, 42^.

Bifidobacteria are specialized towards carbohydrate utilization as reflected by the fact that ~10% of the protein-encoding genes in the *B. breve* UCC2003 genome falls into the functional category devoted to carbohydrate transport and metabolism^29, 53, 57–60^. Among the carbohydrate utilization capabilities identified as essential in *B. breve* UCC2003 and shared with the *Bifidobacterium* core genome, a significant proportion is dedicated to central carbon metabolism (i.e. the bifid shunt and the partially present citric acid cycle) and associated energy metabolism (e.g. pyruvate and pentose phosphate pathways), which also generates important intermediates for biosynthetic purposes (e.g. for the biosynthesis of peptidoglycan, amino acids, fatty acids and nucleotides) (Table 1 and Supplementary Table S2).

Differential niche-adaptation of particular strains to specific environments, may also account for the existence of strain-specific essential genes, not shared in a broader core-genome context^53, 61^. For instance, among the *B. breve* UCC2003 essential genes that are not part of either the *B. breve* or *Bifidobacterium* core genome, various predicted defence mechanisms are included such as DNA methylases and toxin-antitoxin systems. DNA methylases in *B. breve* UCC2003 (encoded by genes BbrI, BbrII and BbrIII) have previously been identified as part of restriction-modification systems, which represent one of the defence barriers against invading DNA, such as bacteriophages^11^. These three methylase-encoding genes were found to be essential in our analysis, thereby confirming that the corresponding restriction enzymes are active in *B. breve* UCC2003. This represents a novel finding since previous experiments by our research team could only prove activity of one of these endonucleases (BbrIII) encoded by this strain^11^. This was most likely due to limited knowledge on the target sequences or on specific mechanisms of reaction on the endonucleases encoded by BbrI and BbrII^11, 62^. Regarding toxin-antitoxin systems, they have not been thoroughly characterized in either *B. breve* or *Bifidobacterium*, however, they are typically associated with mobile genetic elements. Interestingly, in the particular case of this study, we were able to localize as non-core, yet essential, the antitoxin component of a presumptive toxin-antitoxin system (encoded by Bbr_1066 and Bbr_1067), therefore suggesting the corresponding toxin component is active. Indeed, another chromosomal region (located between chromosome positions 907740 and 907450), which had escaped previous ORF detection and annotation efforts, and displaying homology to a toxin-antitoxin module harbours uneven transposon insertions, pointing towards the existence of a second active toxin-antitoxin system in the chromosome of this strain.

In addition, genes essential for bifidobacterial persistence in their natural environment, represented by the gastrointestinal tract, but not required for *in vitro* survival are expected to be present across all bifidobacterial representatives, but may not be among the candidate essential genes delineated in this work. Examples of this are the genes encoding Tad pili or those responsible for autoinducer-2 production^29, 63^, mutants of which were present in the library. Mutations in other *B. breve* UCC2003 functions essential for intestinal colonization in mice, like the non-core gene cluster involved in the biosynthesis of exopolysaccharide (EPS cluster 2)^43^, were also present in the library. Indeed, further research is required to discern the genes that are required for successful gut colonization of *B. breve* UCC2003 (as opposed to being essential for growth under laboratory conditions).

### Comparison between *B. breve* UCC2003 essential functions and those found for other (gut) bacteria

**Figure 4:**
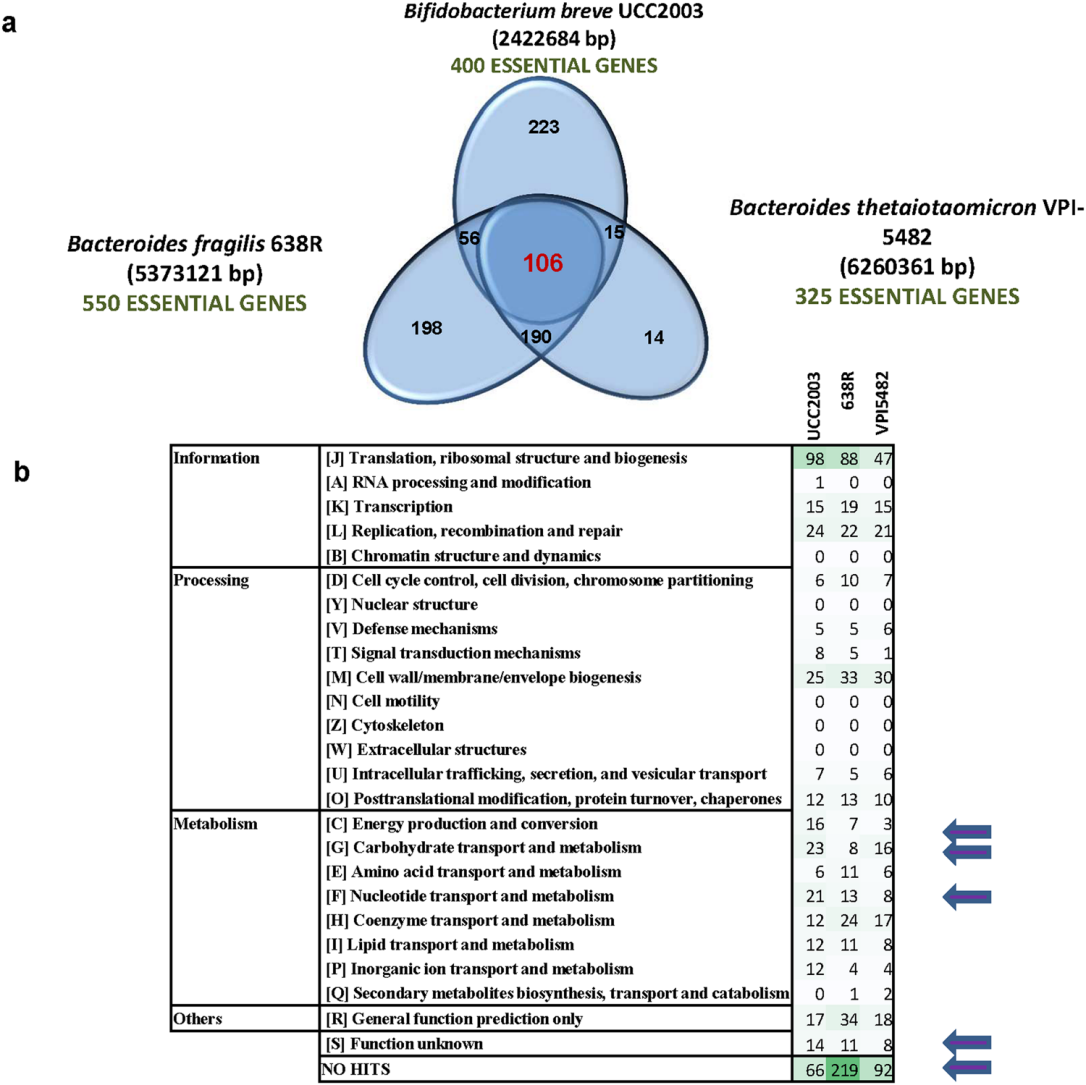
(**a**) Venn Diagram representing the overlap of essential genes determined by transposon sequencing in *B. breve* UCC2003, *Bacteroides fragilis* 638 R and *Bacteroides thetaiotaomicron* VPI-5482. (**b**) Distribution per COG functional classification of essential genes in the three compared taxa.

In order to obtain further information on essential metabolic pathways specific for bifidobacteria and to understand their specific *in vitro* growth requirements, the 400 deduced proteins encoded by the identified essential genes of *B. breve* UCC2003 were compared to proteins corresponding to essential gene surveys reported for other bacteria. *Bacteroides thetaiotaomicron* and *Bacteroides fragilis* are the closest bacteria to bifidobacteria, that have previously been surveyed for essential genes by means of transposon sequencing approaches^18, 32^ (Supplementary Table S5). Although being phylogenetically distant from bifidobacteria, they share the same environmental niche as inhabitants of the human gut. Thus blast-based analysis were used to establish gene orthology between *Bacteroides* and *Bifidobacterium*, and to compare the essential gene compilation detected using the TraDIS approach for these gut bacteria. Despite the limitations of blast-based alignments to identify orthologs accross phyla, as compared to other alignment tools (eg Hidden Markov Model based tools), this analysis revealed that a core set of 106 genes was found essential for these three bacteria, including many housekeeping functions (e.g. DNA maintenance and replication, cell wall and membrane biogenesis and ribosomal and transcriptional machinery). Furthermore the analysis revealed that 223 predicted essential genes in *B. breve* UCC2003 were not detected by TraDIS analysis in *Bacteroides* representatives (Fig. 4a; Supplementary Table S5).

A comparison of the COG classification distribution of essential genes in the three compared taxa (Fig. 4b), showed that *Bifidobacterium* essential genes are particularly enriched in carbohydrate utilization capabilities, nucleotide transport and metabolism, and energy production and conversion. Bifidobacteria are specialized in the utilization of a range of plant- and host-derived carbohydrates, and can rapidly switch their metabolism to adjust to fluctuations in nutrient availability in their natural environment, thus it is not surprising that they have adopted an intricate network of carbohydrate utilization pathways^53, 64^.

Since the three compared TraDIS analyses were performed on libraries with very different coverages, it may be that some essential genes escaped the TraDIS-based predictions. Therefore, a comparative genomic analysis was performed to compare the *Bifidobacterium* predicted essential genes and the two *Bacteroides* genomes. This analysis showed that 126 essential genes of *B. breve* UCC2003 do not exhibit sequence homology to any gene in the two analysed *Bacteroides* genomes, thus constituting bifidobacterial unique genes (Supplementary Table S6). These genes are also part of the *Bifidobacterium* single copy core genome and include functions related to central carbon metabolism, such as the *Bifidobacterium*-specific enzyme fructose-6-phosphoketolase (Bbr_0776) (Supplementary Table S6). A cluster encoding a predicted iron-transport system was found among the *B. breve* UCC2003 specific essential functions (Bbr_0221 to Bbr_0223)^65^. While iron is an essential cofactor for all living bacteria specially anaerobes, the fact that orthologous genes do not appear essential in *Bacteroides* despite their key function in the maintenance of cell homeostasis might either indicate that *Bacteroides* encode additional genes that can compensate the absence of one of the genes or that this systems performs other still undefined essential functions in *B. breve* UCC2003.

Moreover, the essential genes of *B. breve* UCC2003 that do not exhibit homology with the two *Bacteroides* essential gene sets include a relatively high proportion of genes, which encode proteins of unknown functions or for which only general functional predictions can be obtained (46%) (NB. the overall *B. breve* UCC2003 essential gene set contains 15% genes whose products are predicted to have either an unknown or very general function). This highlights that the biological functions of a substantial proportion of essential bifidobacterial genes are still unknown. Indeed, a recent report on the synthetic construction of a minimum *Mycoplasma* genome required the inclusion of 149 (32%) genes of unknown function^41^. These findings and the high proportion of essential hypothetical functions in bifidobacteria emphasize the importance of studying genes encoding hypothetical proteins, particularly because the conclusions of many (microbiome) studies heavily rely on functional predictions.

Finally, it should be mentioned that 29% of the 126 *B. breve* UCC2003 essential genes that do not have homologs in the two analysed *Bacteroides* genomes, represent putative secreted, cell surface-associated proteins or functions that have a direct effect on bacterial surface structure and properties (Supplementary Table S6). These may be crucial in environmental sensing and adaptation, and/or microbe-host interactions, and therefore further investigation into the functionality of hypothetical secreted proteins in bifidobacteria is warranted^66^.

### Conclusions

We identified the genes essential for growth of a commensal *Bifidobacterium* strain under *in vitro* growth conditions, based on a random mutagenesis approach coupled to simultaneous mapping of the disruption point for ~46,000 different insertion sites. Comparison to *Bifidobacterium* and *B. breve* core-genomes revealed significant overlap with our identified candidate essential gene set, especially at single-copy core genes. Essential genes not shared with the *Bifidobacterium* core genome may either represent strain-specific adaptive traits or functions required under specific environmental conditions such as those mimicking food or intestinal environments. In addition, comparison between essential gene sets in other gut commensals, revealed *B. breve* UCC2003-specific essential functions, which upon further study will contribute to a better comprehension of bifidobacterial physiology and evolution. The current work provides a fundamental framework to gain functional insights into bifidobacterial molecular traits at a genome-wide level. Expanding identification and tracking of disrupted genes in collections of bifidobacterial mutants under specific environmental conditions will assist functional annotation in this genus and contribute to a better definition of the minimal bifidobacterial genome. Such knowledge in turn will help to depict a more accurate definition of the bifidobacterial lineage. Furthermore, this work allowed identifying a set of essential functions not previously characterized in bifidobacteria including a range of hypothetical and secreted proteins, the EPS cluster 1 and several orphan ATPases, among others, which represent interesting targets for further studies.

## Methods

### Bacterial strains and culture conditions

*Bifidobacterium breve* UCC2003 was routinely grown anaerobically at 37 °C on RCM (Oxoid) or De Man Rogosa Sharpe (mMRS) made from first principles^67^, and supplemented with 0.05% L-cysteine and appropriate carbon sources. *Escherichia coli* EC101 cells were grown on LB supplemented with tetracycline (10 μg ml^−1^) or kanamycin (50 μg ml^−1^) for selection of plasmid-containing cells. *B. breve* UCC2003 transposon mutants were routinely grown on RCM supplemented with 0.5% ribose and 10 μg ml^−1^ tetracycline, or mMRS supplemented with 0.5% L-cysteine-HCl and appropriate carbon sources as indicated. D-ribose, D-glucose, starch (potato-derived) and D-lactose were purchased from Sigma.

### Creation of second generation Tn5 based transposon, transposome assembly and library construction

A second generation tetracycline resistant transposon was created essentially as previously described^16^. In order to make the transposon library compatible with Transposon Mediated Differential Hybridization (TMDH) approaches, outwards facing T7 promoters were included at the ends of a tetracycline-resistant Tn-*5* transposon. For this purpose, Fw-Tet-pMOD2T7 and Rev-Tet-pMOD2T7 primers (Table 2) were used to amplify a tetracycline resistance cassette, *tetW*, from pAM5 plasmid DNA^68^. This PCR product was digested with XbaI and SphI, and subsequently ligated to similarly digested pMOD2 (Epicentre Biotechnology) to yield pMOD2-tetT7.

**Table 2:** Oligonucleotides and plasmids used in this work.

Name	Sequence	Reference
**Transposon generation**		
pMOD2	Source of Tn*5* terminal ends, AmpR	Epicentre Biotechnology
pMOD2-tetT7	tetR pMOD2 derivative, containing outwards facing T7 promoters at both transposon ends	This work
Fw-Tet-pMOD2T7	CGCTAGTCTAGACCCTATAGTGAGTCGTATTAGCTCATGTACGGTAAGGAG	This work
Rev-Tet-pMOD2T7	CGCTAGGCATGCCCCTATAGTGAGTCGTATTAGCAAAACCCTCGGTCGGTCTGACCGGGGGTTTTGATTACATTACCTTCTGAAACATATGGC	This work
pMOD < MCS > Fw	ATTCAGGCTGCGCAACTGT	Epicentre Biotechnology
pMOD < MCS > Rev	GTCAGTGAGCGAGGAAGCGGAAG	Epicentre Biotechnology
**TraDIS sequencing approach**		
5′ PCR enrichment primer sequence	AATGATACGGCGACCACCGAGATCTACACAATTCGAGCCAATATGCGAGAACACCCG	This work
3′ PCR enrichment primer sequence	AATGATACGGCGACCACCGAGATCTACACATGCAAGCTTGCCAACGACTACGCACTAGC	This work
5′ Sequencing primer sequence	ACCCGAGAAAATTCATCGATGATGGTTGAGATGTGTA	This work
3′ Sequencing primer sequence	ACCCGAGAAAATTCATCGATGATGGTTGAGATGTGTA	This work

^a^Restriction sites are underlined.

The Tn*5*-derived transposon section was then PCR amplified from pMOD2-tetT7 by employing primer combination pMOD < MCS > Fw and pMOD < MCS > Rev (Table 2). The generated PCR product was purified using High pure PCR purification columns (Roche), and transposon ends were pruned by restriction with PshAI. Then DNA was purified and concentrated to 400 ng μl^−1^ before being assembled with purified Tn*5* transposase according to manufacturer’s instructions (Epicentre Biotechnology). About 120 independent electroporation reactions were performed using *B. breve* UCC2003 competent cells, freshly prepared for transformation purposes as previously described^16^. Transformation mixes were spread plated onto RCA agar supplemented with 0.5% ribose and 10 μg ml^−1^ tetracycline, and plates were incubated for 2–3 days anaerobically at 37 °C. In order to create a pool of mutants, plates were flooded with 1 ml of RCM supplemented with 0.5% ribose, 30% glycerol and 10 μg ml^−1^ tetracycline, and clones were scraped from the plates using disposable sterile spreaders. These bacterial suspensions were stored at −80 °C. A master stock was created by pooling the mutants originated from multiple independent experiments.

### DNA isolation and adaptation of TraDIS sequencing and gene essentiality prediction

DNA isolation from the pool of mutants was performed according to previously described procedures^69^. 2 μg DNA was sequenced on a HiSeq2500 platform (Illumina) as previously described^30^, using TraDIS-specific primers designed in this study (see Table 2). Briefly, a read length cut-off of 42 bp after trimming 10 bp off the Tn was used before mapping. Mapping was performed using SMALT v 0.7.6 implemented in Bio-Tradis pipeline using a base PHRED quality threshold of 30 (threshold of 96% of exactly matching nucleotides as a fraction of the read length). No mismatches of reads were allowed in the mapping. Then, gene essentiality was established following a previously described method^30^. Then, transposon insertions located in the 3′ end of a given gene (representing 10% of the gene sequence length) were first excluded, since they do not necessarily inactivate the encoded protein. Then, the insertion index (number of insertion sites per gene length) was calculated for each gene and the distribution of insertion indexes was assessed as being bimodal. Gamma distributions of such indexes were then fitted to the putative essential (mode = 0) and non-essential peaks of the empirical distribution. Log2-likelihood ratios (LLR) were also retrieved between the fitted distributions. Gene essentiality was established based on LLR < −2, with essentiality cut-off set at an insertion index of 0.0015. Genes were classified as non-essential with LLR > 2 and an insertion index cut-off of 0.0021. Insertion indexes falling between these two values were classified as “ambiguous” (Supplementary Figure S4).

### Comparative analysis and core genome predictions

Comparative analyses at species and genus level were conducted using an all-against-all, bi-directional BLASTP alignment^70^ (cut-off: E-value 0.0001, with at least 50% identity across at least 50% of either protein sequence). The BLAST output was then clustered into functionally-related protein families using the Markov Cluster Algorithm (MCL) implemented in the mclblastline pipeline v12–0678^71^. Members of the core-genome were selected based on their presence in all strains and a filter to distinguish orthologues (single-copy) from paralogues (multiple copy) was applied. Members of the core-genome were defined as genes present in each *B. breve* representative. For the comparative analyses extended at genus level, since the fully sequenced *B. breve* UCC2003 was compared with draft genome sequences, we decided to apply an extended core-genome that included genes present in at least 80% of the sequences (in order to exclude the possibility that genes not sequenced were considered absent).

The same BLAST-based comparative analysis was used to compare the *Bifidobacterium breve* UCC2003 essential genes with those found in other (gut) bacteria (e.g. *Bacteroides*). Coding sequences of *B. breve* UCC2003, *Bacteroides fragilis* 638 R and *Bacteroides thetaiotaomicron* VPI-5482 were compared using reciprocal BLASTP alignment^70^ and homologous genes were selected using at least 20% identity across at least 50% of either protein sequence of significant BLAST hits (cut-off: E-value 0.0001) using the MCL algorithm. The information obtained by this clustering was employed to determine the overlap between the essential genes in each TraDIS library and visualized in a Venn diagram and heatmap.

### Functional classification of essential genes and metabolic pathway reconstruction

All *B. breve* UCC2003 genes were functionally classified using a combination of COG category assignment^72^ based on significant BLASTP^70^ alignment against COG database (cut-off: E-value 0.0001, with at least 50% identity across at least 50% of either protein sequence) and refined with the online available information on *B. breve* UCC2003 KEGG pathways (http://www.genome.jp/kegg). The results of this functional classification assignment were then summarized in heatmap (Fig. 3b). The same BLAST-based approach was used to retrieve the COG classification of the essential genes described in other bacteria (belonging to the phylum *Bacteroides*) to be compared with *B. breve* UCC2003 (see above).

## Electronic supplementary material

Supplementary Material

Supplementary Dataset 1

Supplementary Dataset 2

Supplementary Dataset 4

Supplementary Dataset 5

Supplementary Dataset 6
