# Supplementary material for: The essential genomic landscape of the commensal Bifidobacterium breve UCC2003
Source: Sci Rep. 2017 Jul 17;7:5648. doi: 10.1038/s41598-017-05795-y (PMC5514069; doi:10.1038/s41598-017-05795-y)
Supplement: Supplementary file 1 — Supplementary Material [file 41598_2017_5795_MOESM1_ESM.doc]

**Supplementary Material**

**The essential genomic landscape of the commensal *Bifidobacterium breve* UCC2003**

Lorena Ruiz^1ᵻ*^, Francesca Bottacini^1*^, Christine J Boinett^2^, Amy K Cain^2^, Mary O’Connell-Motherway^1^, Trevor D Lawley^2^, Douwe van Sinderen^1^

^1^School of Microbiology and APC Microbiome Institute, National University of Ireland, Cork, Western Road, Ireland

^2^Wellcome Trust Sanger Institute, Hinxton, Cambridge, UK

^ᵻ^Current address: Department of Nutrition, Bromatology and Food Technology, Complutense University, Avda Puerta de Hierro s/n, 28040 Madrid, Spain

*These authors contributed equally to this work

Lorena Ruiz: lorenargar@gmail.com

Francesca Bottacini: francesca.bottacini@ucc.ie

Christine Boinett: cb19@sanger.ac.uk

Amy Cain: ac19@sanger.ac.uk

Mary O'Connell-Motherway: m.oconnellmotherway@ucc.ie

Trevor D Lawley: tl2@sanger.ac.uk

Douwe van Sinderen: d.vansinderen@ucc.ie

**Supplementary Figure S1.**

Rarefaction curve of the transposon mutant library performed using Past3 analysis software (https://folk.uio.no/ohammer/past) with 95 % of confidence. The obtained graph showing saturation of the sampled sites, indicating that essentially all genes of *B. breve* UCC2003 that could be mutated were hit.


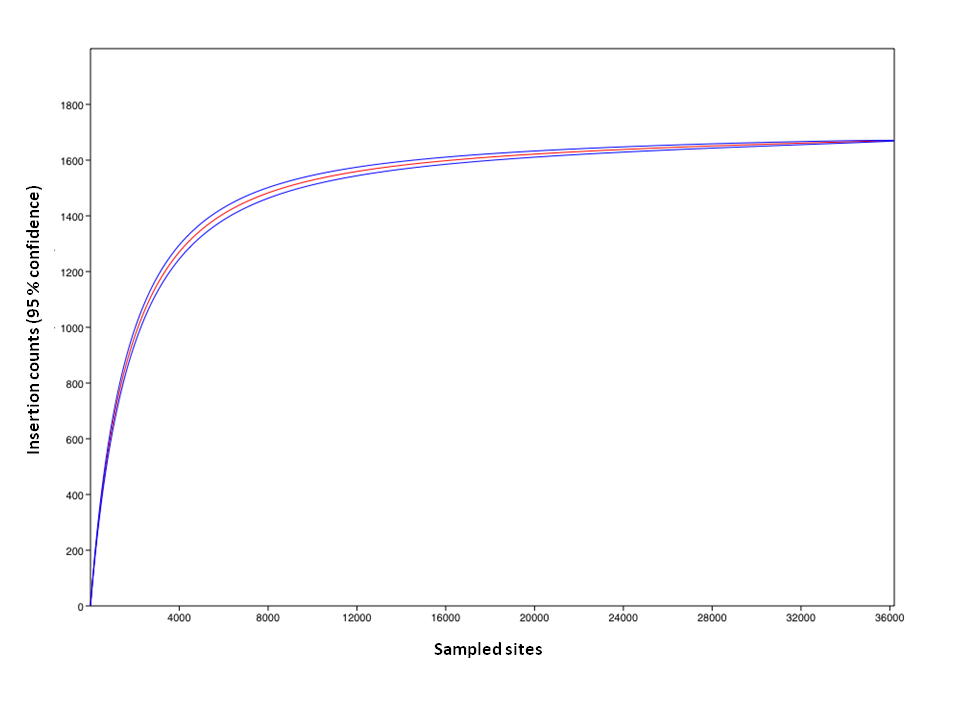


**Supplementary Figure S2.**

A. Graphical representation of the average insertions per gene observed across 8 sections of the *B. brev*e chromosome. Locus tag of genes flanking these 8 regions is also indicated. Dashed lines indicate the average insertion index observed between Reg1-Reg8 in blue (proximal to the origin of replication) and Reg4-Reg5 in red (proximal to the *ter* region).

B. Schematic representation of the tetracycline resistance-conferring tn5 transposon used in this work.


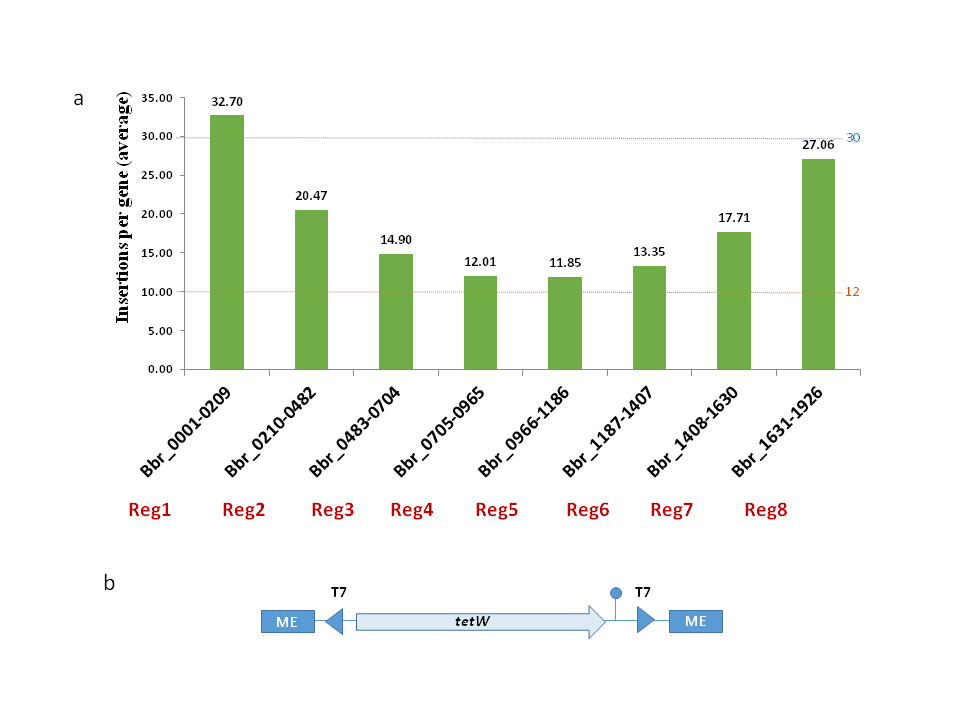


**Supplementary Figure S3.**

A. Graphical representation of insertion index and GC content in *B. breve* UCC2003. EPS cluster 2 region showing the lowest GC content in the genome is also indicated with relative flanking genes.

B. Histogram representing increased insertion index between genes showing low GC content in the EPS region 2.


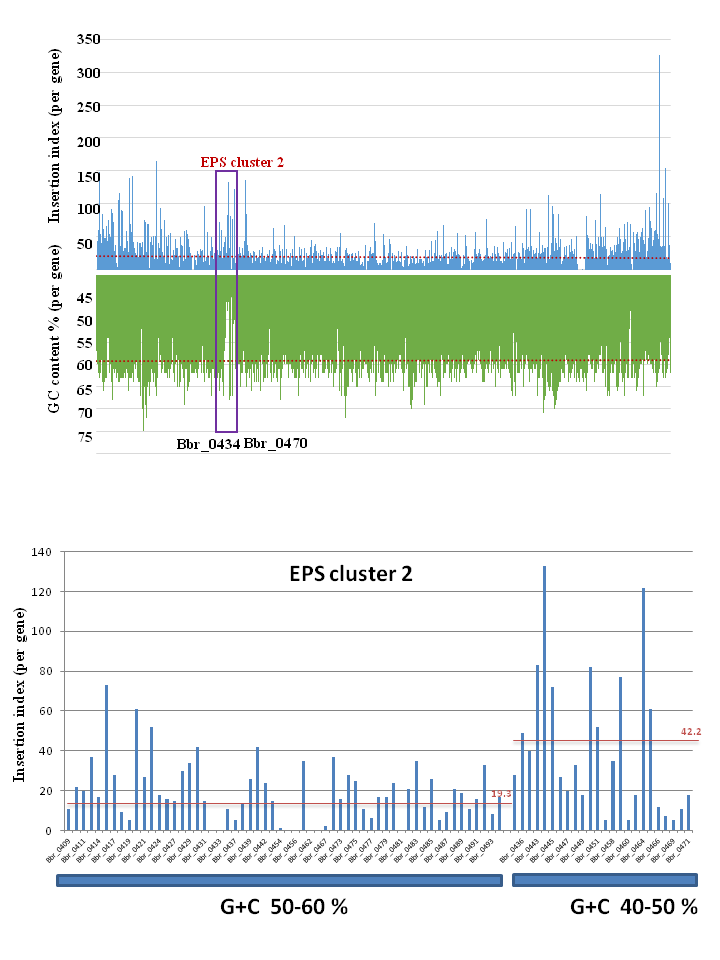


**Supplementary Figure S4.**

Histogram showing gamma fit distribution of insertion indices. Either side of the red vertical lines, give the essential genes (mode at 0) and non-essential genes (right of the second red vertical line with cut-offs of 0.0043 and 0.0059, respectively. The region between the red vertical lines was denoted as ambiguous genes.


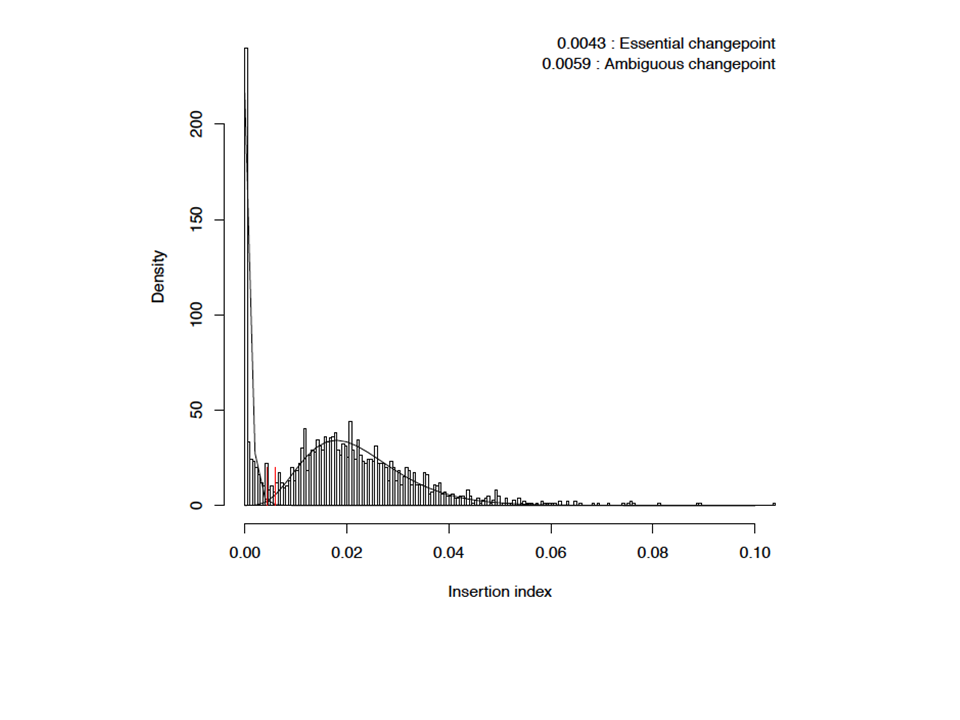


**Supplementary Table S1**

Full list of mapped insertions within *B. breve* UCC2003 predicted genes. Ins_count indicate the number of transposon insertion disruption points within each gene.

**Supplementary Table S2.**

List of TraDIS predicted essential genes in *B. breve* UCC2003. Functional categories based on KEGG annotations are indicated.

**Supplementary Table S3.**

List of genomes used to predict the *Bifidobacterium* and *Bifidobacterium breve* core and variable genomes. Strain origin and accession numbers are indicated.

| **Number** | **Bif genomes** | **Origin** | **Genbank ID** |
| --- | --- | --- | --- |
| 1 | ***Bifidobacterium actinocoloniiforme* DSM 22766** | Bumblebee digestive tract | GYK00000000 |
| 2 | ***Bifidobacterium adolescentis* ATCC 15703** | Adult faeces | AP009256.1 |
| 3 | ***Bifidobacterium angulatum* LMG 11039** | Adult faeces | JGYL00000000 |
| 4 | ***Bifidobacterium animalis* subsp. *animalis* LMG 10508** | Rat faeces | JGYM00000000 |
| 5 | ***Bifidobacterium animalis* subsp. *lactis* DSM 10140** | Fermented milk | CP001606.1 |
| 6 | ***Bifidobacterium asteroides* LMG 10735** | Bee intestine | CP003325.1 |
| 7 | ***Bifidobacterium biavatii* DSM 23969** | Tamarin faeces | JGYN00000000 |
| 8 | ***Bifidobacterium bifidum* LMG 11041** | Infant faeces | JGYO00000000 |
| 9 | ***Bifidobacterium bohemicum* DSM 22767** | Bumblebee digestive tract | JGYP00000000 |
| 10 | ***Bifidobacterium bombi* DSM 19703** | Bumblebee digestive tract | ATLK00000000 |
| 11 | ***Bifidobacterium boum* LMG 10736** | Bovine rumen | JGYQ00000000 |
| 12 | ***Bifidobacterium breve* UCC2003** | Infant faeces | CP000303 |
| 13 | ***Bifidobacterium callitrichos* DSM 23973** | Marmoset faeces | JGYS00000000 |
| 14 | ***Bifidobacterium catenulatum* LMG 11043** | Adult faeces | JGYT00000000 |
| 15 | ***Bifidobacterium choerinum* LMG 10510** | Piglet faeces | JGYU00000000 |
| 16 | ***Bifidobacterium coryneforme* LMG 18911** | Bee intestine | CP007287 |
| 17 | ***Bifidobacterium crudilactis* LMG 23609** | Raw milk cheese | JHAL00000000 |
| 18 | ***Bifidobacterium cuniculi* LMG 10738** | Rabbit faeces | GYV00000000 |
| 19 | ***Bifidobacterium dentium* LMG 11045** | Oral cavity | CP001750.1 |
| 20 | ***Bifidobacterium gallicum* LMG 11596** | Human faeces | JGYW00000000 |
| 21 | ***Bifidobacterium gallinarum* LMG 11586** | Chicken caecum | JGYX00000000 |
| 22 | ***Bifidobacterium indicum* LMG 11587** | Bee intestine | CP006018 |
| 23 | ***Bifidobacterium kashiwanohense* DSM 21854** | Infant faeces | JGYY00000000 |
| 24 | ***Bifidobacterium longum* subsp. *infantis* ATCC 15697** | Infant faeces | AP010889.1 |
| 25 | ***Bifidobacterium longum* subsp. *longum* LMG 13197** | Adult faeces | JGYZ00000000 |
| 26 | ***Bifidobacterium longum* subsp. *suis* LMG 21814** | Piglet faeces | JGZA00000000 |
| 27 | ***Bifidobacterium magnum* LMG 11591** | Rabbit faeces | JGZB00000000 |
| 28 | ***Bifidobacterium merycicum* LMG 11341** | Bovine rumen | JGZC00000000 |
| 29 | ***Bifidobacterium minimum* LMG 11592** | Sewage | JGZD00000000 |
| 30 | ***Bifidobacterium mongoliense* DSM 21395** | Fermented milk | JGZE00000000 |
| 31 | ***Bifidobacterium pseudocatenulatum* LMG 10505** | Infant faeces | JGZF00000000 |
| 32 | ***Bifidobacterium pseudolongum* subsp. *globosum* LMG 11569** | Bovine rumen | JGZG00000000 |
| 33 | ***Bifidobacterium pseudolongum* subsp. *pseudolongum* LMG 11571** | Pig faeces | JGZH00000000 |
| 34 | ***Bifidobacterium psychraerophilum* LMG 21775** | Porcine caecum | JGZI00000000 |
| 35 | ***Bifidobacterium pullorum* LMG 21816** | Chicken faeces | JGZJ00000000 |
| 36 | ***Bifidobacterium reuteri* DSM 23975** | Marmoset faeces | JGZK00000000 |
| 37 | ***Bifidobacterium ruminantium* LMG 21811** | Bovine rumen | JGZL00000000 |
| 38 | ***Bifidobacterium saeculare* LMG 14934** | Rabbit faeces | JGZM00000000 |
| 39 | ***Bifidobacterium sanguini* DSM 23967** | Tamarin faeces | JGZN00000000 |
| 40 | ***Bifidobacterium scardovii* LMG 21589** | Human sources | JGZO00000000 |
| 41 | ***Bifidobacterium stellenboschense* DSM 23968** | Tamarin faeces | JGZP00000000 |
| 42 | ***Bifidobacterium stercoris* DSM 24849** | Adult faeces | JGZQ00000000 |
| 43 | ***Bifidobacterium subtile* LMG 11597** | Sewage | JGZR00000000 |
| 44 | ***Bifidobacterium thermacidophilum* subsp. *porcinum* LMG 21689** | Piglet faeces | JGZT00000000 |
| 45 | ***Bifidobacterium thermacidophilum* subsp. *thermoacidophilum* LMG 21395** | Anaerobic digester | JGZT00000000 |
| 46 | ***Bifidobacterium thermophilum* JCM 1207** | Piglet faeces | JGZV00000000 |
| 47 | ***Bifidobacterium tsurumiense* JCM 13495** | Hamster dental plaque | JGZU00000000 |

**Supplementary Table S4.**

Presence/absence of *B. breve* UCC2003 predicted genes with the predicted *Bifidobacterium* and *B. breve* core genomes.

**Supplementary Table S5.**

Predicted essential genes in *B. breve* UCC2003 compared to reported essential genes from *Bacteroides fragilis* 638R and *Bacteroides thetaiotaomicron* VPI-5482.

**Supplementary Table S6.**

Functional classification of *B. breve* UCC2003 essential genes not sharing homologs in the essential gene lists predicted for *Bacteroides fragilis* 638R and *Bacteroides thetaiotaomicron* VPI-5482.
